# An *in vitro* study of the effects of respiratory circuit setup and parameters on aerosol delivery during mechanical ventilation

**DOI:** 10.3389/fmed.2023.1307301

**Published:** 2024-01-24

**Authors:** Leanne Reilly, Marc Mac Giolla Eain, Sarah Murphy, Andrew O’Sullivan, Mary Joyce, Ronan MacLoughlin

**Affiliations:** ^1^Research and Development, Science and Emerging Technologies, Aerogen Ltd., Galway Business Park, Galway, Ireland; ^2^School of Pharmacy & Biomolecular Science, Royal College of Surgeons in Ireland, Dublin, Ireland; ^3^School of Pharmacy and Pharmaceutical Sciences, Trinty College, Dublin, Ireland

**Keywords:** vibrating mesh nebuliser, mechanical ventilation, suction catheter, adult, paediatric, aerosol drug delivery, drug administration

## Abstract

**Introduction:**

Aerosol therapy is often prescribed concurrently during invasive mechanical ventilation (IMV). This study determines the effects of nebuliser position, circuit humidification source, and most importantly, lung health on the delivery of aerosol in simulated adult and paediatric IMV patients. Furthermore, the influence of closed suction catheters on aerosol delivery is also addressed.

**Methods:**

A vibrating mesh nebuliser was used to deliver Albuterol to simulated adult and paediatric IMV patients with differing states of lung health. Four different nebuliser positions and two types of humidification were analysed. Closed suction catheter mounts, a mainstay in IMV therapy, were incorporated into the circuits. The mean ± SD dose of aerosol (%) was assayed from a filter at the distal end of the endotracheal tube.

**Results:**

Nebuliser placement and circuit humidification source had no effect on the delivered dose (%) in adults, yet both significantly did in the simulated paediatric patients. The use of closed suction catheter mounts significantly reduced the delivered dose (%) in adults but not in paediatric patients. A simulated healthy lung state generated the largest delivered dose (%), irrespective of nebuliser position in the adult. However, different lung health and nebuliser positions yielded higher delivered doses (%) in paediatrics.

**Conclusion:**

Lung health and respiratory circuit composition significantly affect aerosol delivery in both adult and paediatric IMV patients. Nebuliser placement and respiratory circuit humidification source do not affect the delivered dose in adult but do in paediatric IMV patients.

## 1 Introduction

The need for invasive mechanical ventilation (IMV) is common amongst patients admitted to the intensive care unit (ICU). It requires the placement of an artificial airway through a patient’s mouth or nose into the trachea, which is then connected to a ventilator. Invasive mechanical ventilation can stabilise patients with hypoxemic and hypercapnic respiratory failure and reduce the effort required to breathe (1).

Respiratory diseases are amongst the primary causes of admission to the ICU and the subsequent need for IMV (2). The delivery of aerosolised medications concurrently allows for the targeted delivery of high localised drug concentrations (3). The aerosolised delivery of these medications is influenced by a myriad of variables such as drug type, patient, ventilation mode, nebuliser choice and placement, and artificial airway choice (4). There are numerous review papers in the literature that have compared the performance of different aerosol generator types (5–7), their position in the circuit (8–12), and artificial airway choice (13–15) on aerosol drug delivery. While these and other *in vitro* (16) and *in vivo* (17) studies on this topic have greatly increased our understanding of aerosol drug delivery during IMV, there do remain a number of areas not so well understood. These include the use of suctioning systems, circuit humidification source, and lung health in both adult and paediatric models.

In patients undergoing IMV where an endotracheal tube (ETT) is used, they cannot automatically clear airway secretions. As such, the caregiver must perform endotracheal suctioning to maintain airway patency. There are two types of suctioning systems in use: open and closed (18). Closed systems are the preferred option as they do not require the patient to be disconnected from the ventilator; hence, there is no loss in oxygenation or respiratory support (19, 20). There is only a single study that has examined their effect on aerosol drug delivery (21). Only a single delivery position, humidification source and nebuliser type were considered. Despite their widespread use in clinical practice, there is little to no evidence of their affect, if any, on aerosol delivery during IMV.

It has been widely reported that the addition of humidification to the respiratory circuit can reduce the quantity of aerosolised drugs delivered to the lungs; however, a recent review by Fernandez and MacLoughlin (22) found that the effect on clinical outcomes is minimal. The hygroscopic growth of aerosol droplets with humidification is well understood and documented. However, the effects, if any, of the type of humidifier, active or passive, on aerosol drug delivery are not well documented apart from a recent gamma scintigraphy study by Dugernier et al. (23) in ventilated adults in which it was also suggested that the effect of humidification is minimal.

The condition of the patient is a major factor that affects the delivery of aerosolised drugs. Respiratory patients receiving IMV will most likely have reduced lung function. In the diseased lung tidal volumes, resistance, and compliance will be different to that of a healthy lung. For example, in COPD patients, the lung has increased resistance and compliance compared to a healthy lung. However, to date, few studies have examined the influence of lung health, healthy versus diseased lung states, on aerosol delivery. Furthermore, paediatric and infant patients require smaller ventilator circuit tubing and artificial airways and have reduced lung volumes and increased compliance compared to adults. As such, the data obtained from adult clinical studies and bench models cannot be projected to paediatric and infant patients. This study aimed to address these gaps in the literature.

The objective of this study was to examine the effects of nebuliser placement, respiratory circuit humidification type, respiratory circuit components, and lung health on aerosol delivery in simulated adult and paediatric IMV patients and hence provide recommendations on optimal respiratory circuit composition to maximise aerosol delivery.

## 2 Materials and methods

The following section outlines all the materials used to complete this *in vitro* study examining the effects of respiratory circuit setup and parameters on aerosol delivery during simulated mechanical ventilation of adult and paediatric models. All testing was conducted to good laboratory practice and in line with nebuliser test standards in a medical device R&D laboratory. All testing was completed between August and September of 2023.

### 2.1 Aerosol delivery

A vibrating mesh nebuliser (VMN) (Aerogen Solo, Aerogen Ltd., IRE) and a Pro-X controller (Aerogen Ltd., IRE) were used to deliver 2.5 mL of 2.5 mg/2.5 mL of albuterol (Teva Pharmaceuticals, IRE). Aerosol droplet sizing and flow rate were determined by laser diffraction (Malvern Instruments, United Kingdom) (24), and found to be 4.48 ± 0.06 μm and 0.48 ± 0.01 mL/min. The mass of the drug on a capture filter at the distal end of the ETT was quantified using UV-spectrophotometry at 276 nm.

### 2.2 Invasive mechanical ventilation simulation

**Figure 1:**
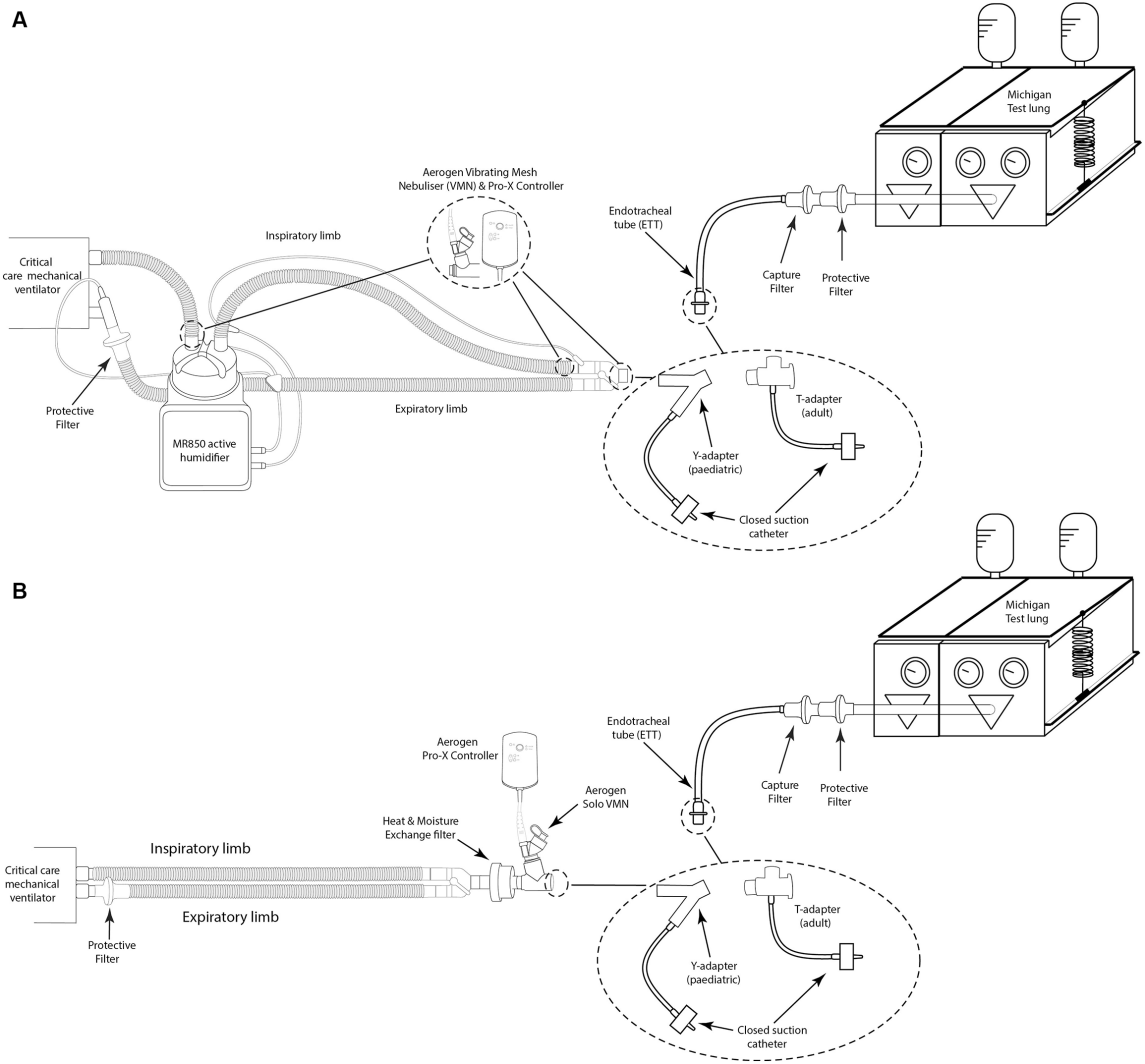
Illustration of simulated invasive mechanical ventilation setup with **(A)** active humidification with and without a closed suction system and **(B)** passive humidification with and without a closed suction system.

An illustration of the experimental setup is presented in Figure 1. A critical care mechanical ventilator (Servo-i, Maquet, GER) incorporating a dual-limb circuit (RT380, F&P, NZ) was utilised with active (MR850, F&P, NZ) or passive humidification (heat moisture exchanger filter (HMEF), PN:1341000S Intersurgical, United Kingdom). Placement of the VMN was varied throughout testing with the nebuliser positioned at the dry side of the humidifier, at the wye, or between the wye and ETT (8.0 mm ID (adult) or 5.5 mm ID (paediatric), Flexicare, United Kingdom). For all testing with the HMEF, the HMEF was connected to the wye and the nebuliser was positioned between the HMEF and the ETT. A capture filter (Respirgard 303, Vyaire, United States) was connected between the ETT and a test lung (Michigan Instruments, USA) and at the ventilator expiratory port.

Lung compliance and resistance on the test lung were altered to simulate different lung states—healthy, obstructive, and restrictive, for both adult and paediatric patient types, see (Table 1) (25, 26). For testing with a closed suction catheter, the T-piece closed suction catheter with Ballard technology (14Fr (adult) or 12Fr (paediatric), Halyard Health, United States) was placed between the wye and ETT. The ventilator settings used to simulate adult (24), and paediatric (30-kg patient) mechanical ventilation (27, 28, 29) are listed in Table 2. The ventilator bias flow for each test condition was recorded at the wye using a flow sensor as previously described in Joyce et al. (30).

**Table 1:** Lung compliance and resistance settings for simulated adult and paediatric models.

Lung State	Adult		Bias Flow (LPM)	Paediatric		Bias flow (LPM)	Compliance (L/cmH_2_O)	Resistance (cmH_2_O/L/s)	Compliance (L/cmH_2_O)	Resistance (cmH_2_O/L/s)
Healthy	0.050	5	2.0	0.030	10	2.1
Obstructive	0.080	20	2.1	0.030	20	2.1
Restrictive	0.040	20	2.0	0.015	10	2.1

**Table 2:** Ventilator settings used to simulated adult and paediatirc patients.

	Adult	Paediatric
Ventilator settings		
*Tidal volume (V_T_) (ml)*	500	240
*Breath rate (BR) (breaths-per-minute, BPM)*	15	25
*Inhalation: Exhalation (I:E) ratio*	1:1	1:2.9
*Tpause (%)*	0	0
*Tinsp. Rise (%)*	5	5
*Trigger Flow (cmH_2_O)*	5	5

### 2.3 Statistical analysis

Results, mean ± standard deviation, for delivered doses are expressed as the percentage of the nominal dose placed in the nebuliser’s medication cup. All testing was conducted in quintuplicate. Statistical analysis was performed using Minitab, V.19 (Minitab LLC, United Kingdom). Two-sample t-tests and one-way ANOVA followed by Tukey *post-hoc* tests were completed to determine the statistical significance of the data. Differences were considered statistically significant when *p* ≤ 0.05.

## 3 Results

### 3.1 Effect of nebuliser position

**Figure 2:**
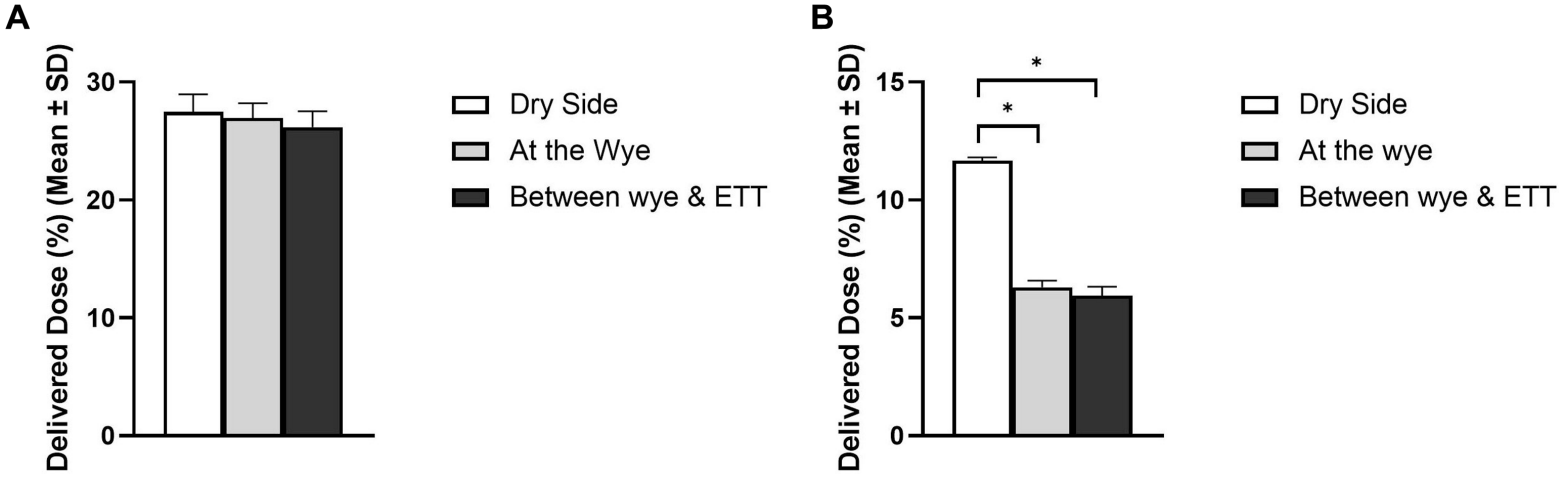
Influence of nebuliser position in the respiratory circuit on aerosol drug delivery in both **(A)** adult and **(B)** paediatric simulated patients. ^*^Denotes *p* ≤ 0.05.

Figure 2 shows the impact of the nebuliser position on aerosol drug delivery during simulated IMV in both (A) adult and (B) paediatric patient types. A healthy lung was simulated in both patient cohorts, and the respiratory circuit did not make use of a closed suction catheter system. In the simulated adult, nebuliser placement had no statistically significant impact on the delivered dose (%) of aerosol, *p* = 0.353. However, in the simulated paediatric patient, nebuliser placement did have a statistically significant impact, *p* = 0.000, with the greatest quantity of aerosolised drug delivered when the nebuliser was placed at the dry side of the humidifier, 11.67 ± 0.13%.

### 3.2 Effect of humidification source

**Figure 3:**
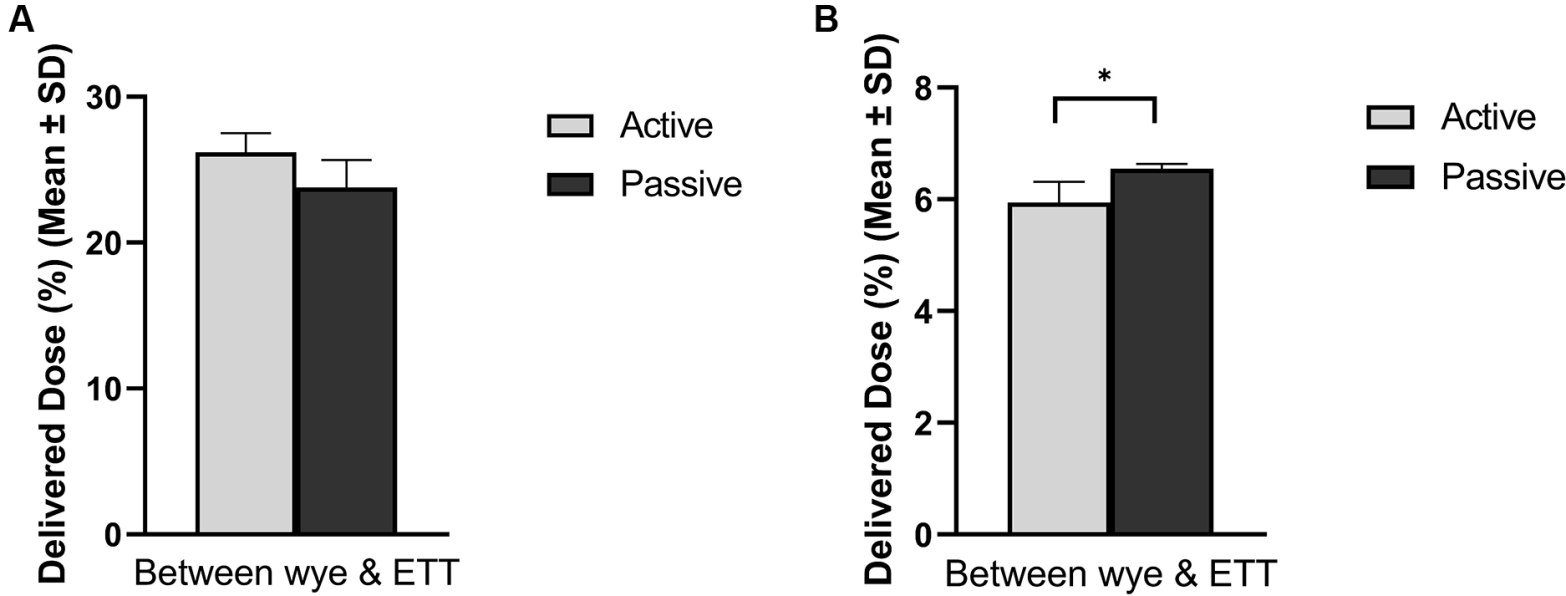
Influence of humidification source on aerosol drug delivery in mechanically ventilated **(A)** adult and **(B)** paediatric simulated patients. ^*^Denotes *p* ≤ 0.05.

Figure 3 compares active and passive humidification sources on aerosol drug delivery in both (A) adult and (B) paediatric patients. A healthy functioning lung was simulated in both patient cohorts, and the respiratory circuit did not make use of a closed suction catheter. In the simulated adult, the humidification source did not have a statistically significant impact, *p* = 0.054. While in the simulated paediatric patient, there was a significant difference in aerosolised drug dose delivered, *p* = 0.024, 5.94 ± 0.37% active and 6.55 ± 0.09% passive humidification.

### 3.3 Effect of catheter suction system

**Figure 4:**
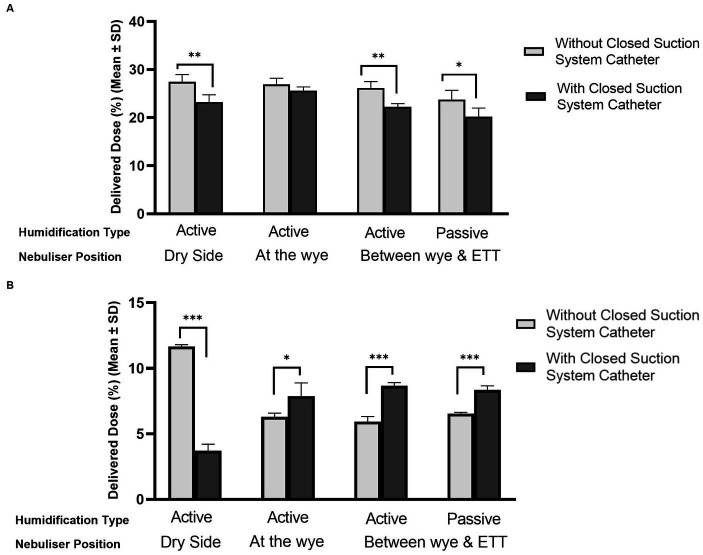
Influence of catheter suction system on aerosol drug delivery in mechanically ventilated **(A)** adult and **(B)** paediatric simulated patients. ^*^Denotes *p* ≤ 0.05, ^**^p ≤ 0.01, and ^***^p ≤ 0.001.

Figure 4 compares the levels of aerosol drug delivery in (A) adult and (B) paediatric IMV patients with and without the presence of closed suction catheter systems in the respiratory circuit. The humidification source and nebuliser position within the respiratory circuit are also accounted for in the plots. A healthy functioning lung was simulated for both patient cohorts. In the simulated adult, the addition of the closed suction catheter system did not have a statistically significant impact on the delivered dose when the nebuliser was placed at the wye and active humidification was in use, *p* = 0.084. However, the addition of the closed suction catheter did significantly reduce the quantity of aerosolised drug delivered for the other nebuliser positions considered in this study, dry side *p* = 0.003, between the wye and the ETT *p* = 0.002 (active) and *p* = 0.017 (passive). When the catheter was used with the simulated paediatric patient (Figure 4B), significantly less drug was delivered to the lung when the nebuliser was positioned at the dry side only (*p* = 0.000). For all other positions and both humidification sources, significantly more drug was delivered to the lung when the closed suction catheter was used, *p* < 0.05.

### 3.4 Effect of lung health

**Figure 5:**
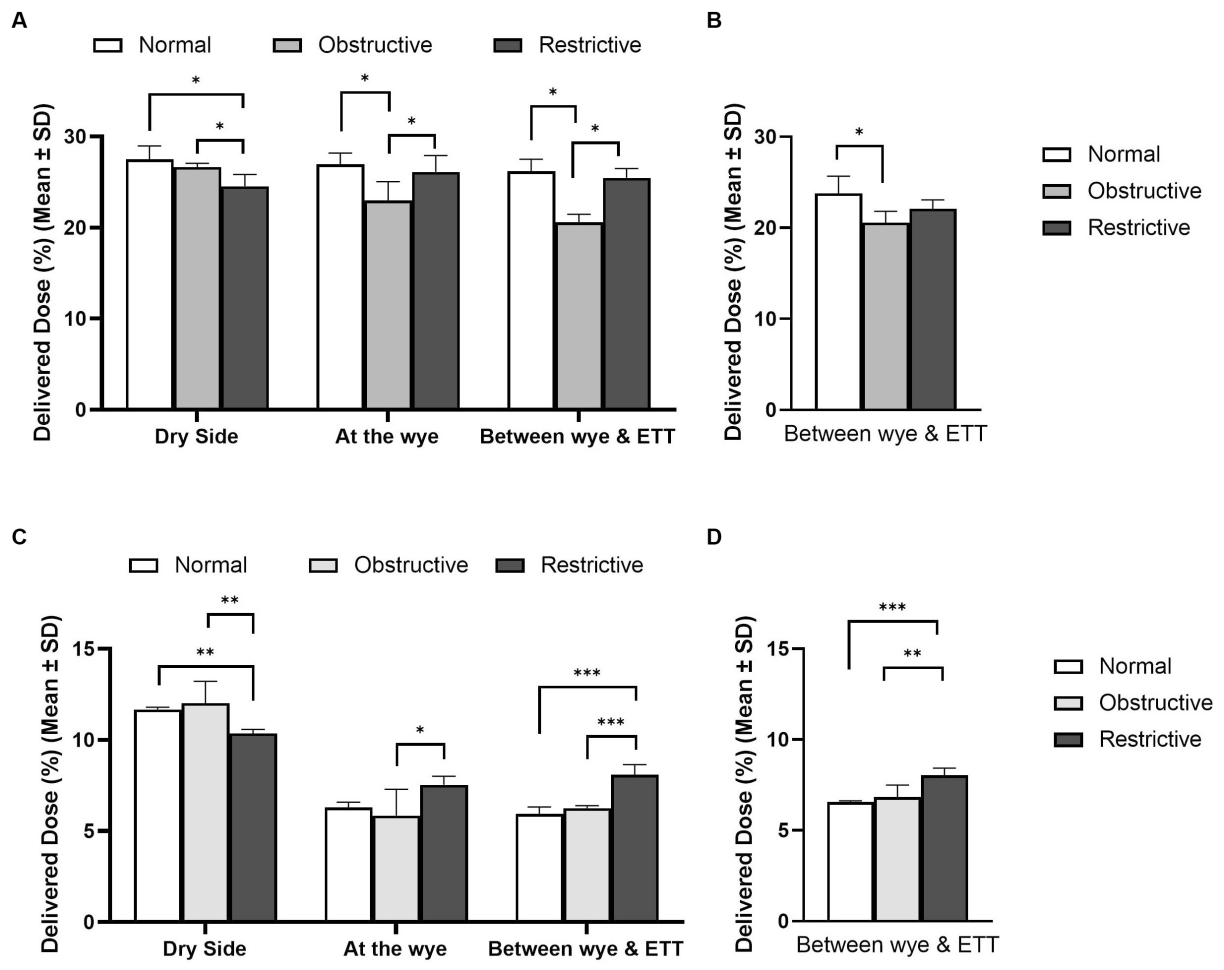
Influence of lung disease on aerosol drug delivery in mechanically ventilated adult, **(A,B)**, and paediatric, **(C,D)**, simulated patients with active **(A,C)** and passive humidification **(B,D)**. ^*^Denotes *p* ≤ 0.05, ^**^p ≤ 0.01 and ^***^p ≤ 0.001.

Figure 5 highlights the influence of lung health on the quantity of aerosol available for delivery in adult, (A) and (B), and paediatric, (C) and (D), patients. The position of the nebuliser and the type of humidification used in the respiratory circuit are also detailed. The respiratory circuits did not incorporate closed suction catheters. In the simulated adult, lung health had a statistically significant effect, p < 0.05, irrespective of nebuliser position and humidification source. Similarly, in the simulated paediatric patients, lung health had a statistically significant effect on the quantity of aerosol delivered to the lungs, *p* < 0.05. However, unlike in the simulated adult, the healthy lung did not receive the largest dose of aerosol. For example, with the nebuliser placed at the wye, the delivered doses were: 6.30 ± 0.28% (healthy), 5.84 ± 1.45% (obstructive), and 7.55 ± 0.48% (restrictive).

## 4 Discussion

This *in vitro* study assessed the effects of nebuliser placement and respiratory circuit humidification source using the most clinically relevant respiratory circuit arrangements encountered in the critical care setting. These circuits included closed suction catheter mounts, which are a mainstay in respiratory circuits. Furthermore, the effects of differing levels of lung health on aerosol drug delivery in both simulated adult and paediatric patients were examined. It was found that nebuliser placement and respiratory circuit humidification had no significant effect on the delivered dose (%) in the simulated adult; however, the opposite was found in the simulated paediatric models. Lung health had a significant effect on the delivered dose in both simulated patient cohorts, irrespective of nebuliser placement and humidification source. A simulated healthy lung received the largest delivered dose (%) in the adult but not in the paediatric model. Analysis of capture filters placed on the ventilator expiratory port showed that significant quantities of aerosol travel to the ventilator during aerosol therapy and are a major source of loss during aerosol therapy.

In the recently published AMIKINHAL study by Ehrmann et al. (31), the potential for optimised aerosol delivery in ventilated patients was clearly demonstrated. As discussed, and evaluated here, the delivered dose may be affected by a myriad of factors. Previous research studies by authors, such as Ari et al. (32), Sidler-Moix et al. (7), Dugernier et al. (33), and Montigaud et al. (34) have studied similar parameters to those presented in this study. However, like-for-like comparisons are not possible due to differences in ventilators, ventilation modes and settings, nebuliser types, drug dosages, simulated patient settings, and lung health. As such, the general trends in this study are discussed in relation to other studies in this field.

In the simulated adult patient, nebuliser placement had no statistically significant effect on the delivered dose of aerosol, *p* = 0.353. In the simulated paediatric patient, nebuliser placement did have a statistically significant effect, *p* = 0.000. The largest delivered dose of aerosol (%) occurred for both patient cohorts when the nebuliser was placed on the dry side of the humidifier, 27.47 ± 1.50% adult and 11.67 ± 0.13% paediatric. Similar observations were also noted in previous studies by Dugernier et al. (10), Anderson et al. (8), Rajendran et al. (35), and Berlinski and Willis (28). Furthermore, nebuliser placement at this position yielded the least amount of aerosol at the ventilator expiratory port, 8.15 ± 0.84% *p* = 0.04 adult and 6.83 ± 0.73% *p* = 0.000, see Supplementary Tables S1, S2. When the VMN is placed proximal to the patient, the generated aerosol is driven down the expiratory port line between inspirations. Hence, the significantly greater quantities of aerosol on the ventilator expiratory port filter when the nebuliser is placed at the wye and between the wye and ETT in both the simulated adult and paediatric patients. This, over 16%, and the smaller ETT tube, tidal volume, and I:E ratio, explain why nebuliser placement has a significant effect on the delivered dose (%) in the simulated paediatric patient and not the simulated adult patient.

It is well understood that the addition of humidification to the respiratory circuit reduces the quantity of aerosol available for inhalation in bench models, see (36). Active humidification is the most common type incorporated into an IMV circuit. There was a statistically significant effect in the simulated paediatric patient, *p* = 0.024, with the use of passive humidification resulting in a greater delivered dose, 6.55 ± 0.09% compared to 5.94 ± 0.37% for active humidification. However, the opposite was noted in the simulated adult, *p* = 0.054, 26.18 ± 1.34% active humidification compared to 23.79 ± 1.88% passive humidification, which agrees with previous reports by Pelosi et al. (37), Iotti et al. (38), and Naughton et al. (39). The addition of an HMEF has been shown to increase circuit resistance, which necessitates an increase in the work of breathing by the patients. It is possible that this increase in work of breathing in the simulated paediatric model generated the larger delivered dose (37, 38).

To the best of the author’s knowledge, this is the first study to assess the impact of closed suction catheter mounts on aerosol delivery from a VMN. The use of closed suction catheters resulted in a lower delivered dose of aerosol, irrespective of nebuliser placement and humidification source in the simulated adult. Williams et al. (21) compared the effects of different closed suction catheter designs and pMDI adapters on aerosol delivery in simulated adult IMV patients. However, no comparison was made to the case when no such a system was used; thus, no conclusions could be made on the effects of the system itself. Unlike in the simulated adult, the use of closed suction catheters resulted in a larger delivered dose in the simulated paediatric patient when the nebuliser was placed at the wye and between the wye and ETT (*p* < 0.05). Given the design differences in the catheter mounts, the longer expiratory phase, 2.9 times that of inspiration, and these particular locations in the circuit, the catheter may function as an aerosol holding chamber. The aerosol bolus accumulates here during exhalation and is then inhaled during the inspiratory phase. This potential build-up would not occur in the simulated adult as the inspiratory and expiratory phases are the same length, 1:1.

Physiologically lung diseases can be categorised as restrictive, such as ARDS, obstructive, such as COPD, or a combined pattern of both (40). Aerosol delivery to lungs where these diseases were simulated was benchmarked against simulated healthy lungs in both adult and paediatric patients, see Table 1. In the simulated adult, the healthy lung received the largest dose of aerosol, irrespective of nebuliser placement and circuit humidification (*p* < 0.05). Comparing aerosol delivery in the diseased lungs, nebuliser placement had a statistically significant effect when active but not passive humidification was incorporated into the respiratory circuit. Hess and Kacmarek (41) compared the effects of lung resistance and compliance and ventilation mode on the delivery of albuterol. The authors found that there was greater aerosol delivery to the lung with a higher resistance and compliance, which agrees with the current study and that of Darquenne (42) when the VMN was placed on the dry side only. It should be noted that the study by Hess and Kacmarek (41) consisted of a different respiratory circuit setup, no ETT, considered only a single nebuliser position, made use of different ventilation parameters, resistances, and compliances. In the simulated paediatric patient, lung health also had a statistically significant effect on aerosol delivery, irrespective of nebuliser placement or circuit humidification (*p* < 0.05). However, unlike in the simulated adult, the simulated healthy paediatric lung did not receive the largest dose at each nebuliser position considered. For instance, when placed at the wye, the simulated restrictive lung received a dose of 7.53 ± 0.48%, whereas the healthy lung was 6.30 ± 0.28%. Given the similarities in compliance and resistance of the different lung states considered in this study (Table 1), it stands to reason that at the different nebuliser locations, different states of lung health could receive larger delivered doses (%). This would again highlight the importance of nebuliser placement in the respiratory circuit in a paediatric IMV patient.

An interesting caveat of this study is the quantity of aerosol collected on the ventilator expiratory port filter. In a circuit with active humidification, the most common type utilised in IMV circuits, this could be as high as 19.39 ± 1.76% in adults and 20.07 ± 1.40% in paediatric patients (Supplementary Tables S1, S2). This is a significant source of loss of the original dose of therapeutic and a potential source of contamination and damage to the ventilator and the environment. The use of HME filters, given their place in the respiratory circuit, eliminates this almost entirely, approximately 0.4% for both patient types, see Supplementary Tables S1, S2.

There are several limitations to this study. The *in vitro* lung doses overestimate the actual dose as the filters do not allow exhalation of the aerosol fraction that is deposited on the filter, but otherwise might have been exhaled from an airway. A recent study by Dugernier et al. (23) suggests that *in vitro* studies, making use of filters overestimate the actual *in vivo* lung dose by approximately 10%. Furthermore, the effects of exhaled heat and humidity were not accounted for in the data. Future research is warranted to better understand the effects, if any, of exhaled heated air through the respiratory circuit on aerosol delivery. Only a single ventilator type and ventilation mode were considered in this study; in an actual critical care setting, the ventilator mode and settings would be adjusted frequently to account for the patient’s lung health. For example, it is a common practice to implement low tidal volume ventilation strategies to prevent ventilator-associated lung injuries and further damage the airways. Further study in this is required to aid in optimising patient treatment. Similarly, only a single type of artificial airway was used; devices such as tracheostomy tubes and supraglottic airways, given their prevalence, warrant research.

## 5 Conclusion

Nebuliser placement and respiratory circuit humidification source had no significant effect on the delivered dose (%) in the simulated adult, while the opposite was found in the simulated paediatric patient. In the simulated adult, lung health was found to influence the delivered dose (%), where the healthy lung received the largest dose (%), irrespective of nebuliser placement and circuit humidification source. In the simulated paediatric model, changes in both lung health and nebuliser placement affected the delivered dose. The incorporation of closed suction catheter mounts, a mainstay in IMV circuits, was found to reduce the delivered dose in the simulated adult. However, in the simulated paediatric patient the opposite was found and was attributed to the extended expiratory phase in the respiratory cycle. The data highlight that adult models cannot be simply scaled to infants and paediatrics. Our findings show that the largest dose of aerosol was delivered when the nebuliser was placed on the dry side of the humidifier and the respiratory circuit incorporated active humidification in both model types and hence the most effective respiratory circuit arrangement.

## Data availability statement

The original contributions presented in the study are included in the article/Supplementary material, further inquiries can be directed to the corresponding author.

## Author contributions

LR: Conceptualization, Formal analysis, Writing – original draft, Writing – review & editing, Data curation, Methodology, Project administration, Validation. MMGE: Conceptualization, Writing – review & editing, Formal analysis, Writing – original draft. SM: Conceptualization, Writing – review & editing, Data curation. AO’S: Conceptualization, Formal analysis, Writing – review & editing. MJ: Conceptualization, Formal analysis, Supervision, Writing – review & editing, Methodology. RM: Conceptualization, Formal analysis, Supervision, Writing – review & editing.
