# Supplementary material for: An in vitro study of the effects of respiratory circuit setup and parameters on aerosol delivery during mechanical ventilation
Source: Front Med (Lausanne). 2024 Jan 24;10:1307301. doi: 10.3389/fmed.2023.1307301 (PMC10847248; doi:10.3389/fmed.2023.1307301)
Supplement: Supplementary file 1 [file Table_1.DOCX]

**Supplementary Tables:**

**Table 1.** Percentage drug delivered to lung and ventilator expiratory inlet for several test scenarios varying nebuliser position, humidification source, use of closed suction system and disease type, during invasive mechanical ventilation in a simulated adult patient. The values represented are mean ± standard deviation for five independent experiments. Differences were considered statistically significant when p ≤ 0.05.

| Factor | Nebuliser Position | Humidification Source | Closed Suction System in Circuit (Yes/No) | Lung health | Lung Dose (%) | p-value | Ventilator Expiratory Port (%) | p-value |
| --- | --- | --- | --- | --- | --- | --- | --- | --- |
| Nebuliser Position | Dry Side | Active | No | Healthy | 27.47 ± 1.50 | 0.353 | 8.15 ± 0.84 | 0.004 |
|  | Wye |  |  |  | 26.96 ± 1.24 |  | 10.14 ± 0.92 |  |
|  | Between wye & ETT |  |  |  | 26.18 ± 1.34 |  | 11.12 ± 1.46 |  |
| Humidification Source | Between wye & ETT | Active | No | Healthy | 26.18 ± 1.34 | 0.054 | 11.12 ± 1.46 | 0.000 |
|  |  | Passive |  |  | 23.79 ± 1.88 |  | 0.45 ± 0.23 |  |
| Closed Suction System | Dry Side | Active | No | Healthy | 27.47 ± 1.50 | 0.003 | 8.15 ± 0.84 | 0.260 |
|  |  |  | Yes |  | 23.26 ± 1.52 |  | 8.67 ± 0.39 |  |
|  | Wye | Active | No |  | 26.96 ± 1.24 | 0.084 | 10.14 ± 0.92 | 0.000 |
|  |  |  | Yes |  | 25.59 ± 0.82 |  | 14.88 ± 0.64 |  |
|  | Between wye & ETT | Active | No |  | 26.02 ± 1.34 | 0.002 | 11.12 ± 1.46 | 0.000 |
|  |  |  | Yes |  | 22.23 ± 0.69 |  | 18.72 ± 1.68 |  |
|  |  | Passive | No |  | 23.65 ± 1.86 | 0.017 | 0.45 ± 0.23 | 0.922 |
|  |  |  | Yes |  | 20.18 ± 1.81 |  | 0.43 ± 0.10 |  |
| Lung health | Dry Side | Active | No | Healthy | 27.47 ± 1.50 | 0.005 | 8.15 ± 0.84 | 0.043 |
|  |  |  |  | Obstructive | 26.64 ± 0.43 |  | 9.23 ± 1.65 |  |
|  |  |  |  | Restrictive | 24.53 ± 1.31 |  | 7.26 ± 0.34 |  |
|  | Wye | Active |  | Healthy | 26.96 ± 1.24 | 0.008 | 10.14 ± 0.92 | 0.033 |
|  |  |  |  | Obstructive | 22.96 ± 2.06 |  | 11.92 ± 1.30 |  |
|  |  |  |  | Restrictive | 26.10 ± 1.03 |  | 11.84 ± 0.88 |  |
|  | Between wye & ETT | Active |  | Healthy | 26.18 ± 1.34 | 0.000 | 11.12 ± 1.46 | 0.000 |
|  |  |  |  | Obstructive | 20.58 ± 0.89 |  | 19.39 ± 1.76 |  |
|  |  |  |  | Restrictive | 25.45 ± 1.03 |  | 17.01 ± 1.96 |  |
|  |  | Passive |  | Healthy | 23.79 ± 1.88 | 0.012 | 0.45 ± 0.23 | 0.003 |
|  |  |  |  | Obstructive | 20.56 ± 1.25 |  | 0.02 ± 0.05 |  |
|  |  |  |  | Restrictive | 22.08 ± 0.98 |  | 0.13 ± 0.14 |  |

**Table 2.** Percentage drug delivered to lung and ventilator expiratory inlet for several test scenarios varying nebuliser position, humidification source, use of closed suction system and disease type, during invasive mechanical ventilation in a simulated paediatric patient. The values represented are mean ± standard deviation for five independent experiments. Differences were considered statistically significant when p ≤ 0.05.

| Factor | Nebuliser Position | Humidification source | Closed Suction System in Circuit (Yes/No) | Lung health | Lung Dose (%) | p-value | Ventilator Expiratory Port (%) | p-value |
| --- | --- | --- | --- | --- | --- | --- | --- | --- |
| Nebuliser Position | Dry Side | Active | No | Healthy | 11.67 ± 0.13 | 0.000 | 6.83 ± 0.73 | 0.000 |
|  | Wye |  |  |  | 6.30 ± 0.28 |  | 16.89 ± 1.92 |  |
|  | Between wye & ETT |  |  |  | 5.94 ± 0.37 |  | 16.59 ± 0.94 |  |
| Humidification source | Between wye & ETT | Active | No | Healthy | 5.94 ± 0.37 | 0.024 | 16.59 ± 0.94 | 0.000 |
|  |  | Passive |  |  | 6.55 ± 0.09 |  | 0.26 ± 0.11 |  |
| Closed Suction System | Dry Side | Active | No | Healthy | 11.67 ± 0.13 | 0.000 | 6.83 ± 0.73 | 0.029 |
|  |  |  | Yes |  | 3.73 ± 0.49 |  | 7.92 ± 0.50 |  |
|  | Wye | Active | No |  | 6.30 ± 0.28 | 0.028 | 16.89 ± 1.92 | 0.046 |
|  |  |  | Yes |  | 7.87 ± 1.01 |  | 19.41 ± 0.42 |  |
|  | Between wye & ETT | Active | No |  | 5.94 ± 0.37 | 0.000 | 16.59 ± 0.94 | 0.002 |
|  |  |  | Yes |  | 8.67 ± 0.24 |  | 20.07 ± 1.40 |  |
|  |  | Passive | No |  | 6.55 ± 0.09 | 0.000 | 0.26 ± 0.11 | 0.000 |
|  |  |  | Yes |  | 8.35 ± 0.32 |  | 2.72 ± 0.05 |  |
| Lung health | Dry Side | Active | No | Healthy | 11.67 ± 0.13 | 0.007 | 6.83 ± 0.73 | 0.082 |
|  |  |  |  | Obstructive | 12.02 ± 1.20 |  | 7.93 ± 1.41 |  |
|  |  |  |  | Restrictive | 10.35 ± 0.23 |  | 6.48 ± 0.48 |  |
|  | Wye | Active |  | Healthy | 6.30 ± 0.28 | 0.030 | 16.89 ± 1.92 | 0.541 |
|  |  |  |  | Obstructive | 5.84 ± 1.45 |  | 16.30 ± 1.36 |  |
|  |  |  |  | Restrictive | 7.53 ± 0.48 |  | 17.42 ± 1.32 |  |
|  | Between wye & ETT | Active |  | Healthy | 5.94 ± 0.37 | 0.000 | 16.59 ± 0.94 | 0.000 |
|  |  |  |  | Obstructive | 6.25 ± 0.15 |  | 16.62 ± 0.44 |  |
|  |  |  |  | Restrictive | 8.09 ±0.55 |  | 11.34 ± 0.48 |  |
|  |  | Passive |  | Healthy | 6.55 ± 0.09 | 0.000 | 0.26 ± 0.11 | 0.000 |
|  |  |  |  | Obstructive | 6.82 ± 0.67 |  | 0.46 ± 0.09 |  |
|  |  |  |  | Restrictive | 8.03 ± 0.40 |  | 0.07 ± 0.05 |  |
